# Applying a network framework to the neurobiology of reading and dyslexia

**DOI:** 10.1186/s11689-018-9251-z

**Published:** 2018-12-13

**Authors:** Stephen K. Bailey, Katherine S. Aboud, Tin Q. Nguyen, Laurie E. Cutting

**Affiliations:** Peabody College, Vanderbilt University, One Magnolia Circle, Nashville, TN USA

**Keywords:** Dyslexia, Brain network, Individual differences, Reading, Language, Functional MRI, Graph theory

## Abstract

**Background:**

There is a substantial literature on the neurobiology of reading and dyslexia. Differences are often described in terms of individual regions or individual cognitive processes. However, there is a growing appreciation that the brain areas subserving reading are nested within larger functional systems, and new network analysis methods may provide greater insight into how reading difficulty arises. Yet, relatively few studies have adopted a principled network-based approach (e.g., connectomics) to studying reading. In this study, we combine data from previous reading literature, connectomics studies, and original data to investigate the relationship between network architecture and reading.

**Methods:**

First, we detailed the distribution of reading-related areas across many resting-state networks using meta-analytic data from NeuroSynth. Then, we tested whether individual differences in modularity, the brain’s tendency to segregate into resting-state networks, are related to reading skill. Finally, we determined whether brain areas that function atypically in dyslexia, as identified by previous meta-analyses, tend to be concentrated in hub regions.

**Results:**

We found that most resting-state networks contributed to the reading network, including those subserving domain-general cognitive skills such as attention and executive function. There was also a positive relationship between the global modularity of an individual’s brain network and reading skill, with the visual, default mode and cingulo-opercular networks showing the highest correlations. Brain areas implicated in dyslexia were also significantly more likely to have a higher participation coefficient (connect to multiple resting-state networks) than other areas.

**Conclusions:**

These results contribute to the growing literature on the relationship between reading and brain network architecture. They suggest that an efficient network organization, i.e., one in which brain areas form cohesive resting-state networks, is important for skilled reading, and that dyslexia can be characterized by abnormal functioning of hub regions that map information between multiple systems. Overall, use of a connectomics framework opens up new possibilities for investigating reading difficulty, especially its commonalities across other neurodevelopmental disorders.

**Electronic supplementary material:**

The online version of this article (10.1186/s11689-018-9251-z) contains supplementary material, which is available to authorized users.

## Background

Reading is a complex cognitive act. To read, individuals must precisely control visual attention, map symbols to phonological representations, extract meaning from words, update mental representations of the text, inhibit unimportant associations, and make appropriate inferences. Consequently, while the most explicit aim of reading instruction and intervention is to build fast and efficient orthographic-phonological mapping, reading difficulty can arise from many sources [1, 2]. To further complicate matters, reading disability is often comorbid with other learning and developmental disorders, such as specific language impairment and attention deficit/hyperactivity disorder [3, 4].

In the past two decades, neuroimaging research has provided valuable insights into the neural mechanisms of typical and atypical reading. Researchers have found that reading co-opts the brain’s visual system to introduce a new input pathway into existing language comprehension circuitry [5]. As text complexity increases, a larger demand is made on attentional systems, and activation becomes more bilateral and widespread [6]. Meta-analyses show that individuals with reading difficulty typically exhibit underactivation in areas responsible for recognizing symbol units, parsing acoustic sounds into phonological units, and binding letters to sounds [7–9]. However, many questions remain regarding the root causes of dyslexia, how to best identify children at risk and the reasons for its high comorbidity with other developmental disorders.

Connectivity-based neuroimaging methods provide an alternative framework to examine reading difficulties. Whereas traditional approaches focus on identifying focal regions of deficit, many learning and psychiatric disorders are characterized, in part, by how brain *networks* behave and interact. In particular, connectomics analyses have shown that the brain exhibits a network configuration which allows for high transferability of information at minimal cost, i.e., a “small-world” network architecture [10]. Two attributes of brain organization have been of special interest: the presence of densely intra-connected *modules*, often called resting-state networks (RSNs) [11], and the existence of a core group of *hub areas* that play an outsize role in conveying information between RSNs [12]. This small-world architecture appears to reach peak efficiency in young adulthood, with younger children exhibiting fewer long-range RSNs [13] and older adults showing a decrease in modularity, especially in higher-order RSNs like the default mode network [14].

Since reading requires rapid interaction and manipulation of disparate cognitive processes, the network framework is an appealing avenue of investigation in reading disorders. Previous research has suggested that the areas responsible for reading do not form an independent system, but are instead distributed across multiple RSNs [15]. There is evidence that the lower modularity within these RSNs (e.g., the default mode network) could be predictive of disorders, including attention deficit hyperactivity disorder [16]. Furthermore, damage to hub areas can cause devastating behavioral effects [17] and may be degenerated in psychiatric and developmental disorders such as schizophrenia, Alzheimer’s disease, and ADHD [18]. Graph theory measures of connectedness within and between RSNs may consequently be related to differences in reading skill. However, while a small number of papers indicate that they may be affected in dyslexia [19, 20], its application in the reading domain has been relatively sparse, with few emergent themes thus far [21]. This is surpising because connectomics data can be procured without using cognitive tasks (which represent a confounding variable) and because they provide a common neurobiological framework for understanding cognitive disorders.

In this paper, we test the hypothesis that an efficient global network architecture is important for skilled reading. First, we establish that areas subserving reading fall into many RSNs, rather than loading onto a single network. Second, we test whether better readers are more likely to have high modularity during rest, i.e., densely intra-connected RSNs. Finally, we determine whether dyslexia, like other psychiatric disorders, disproportionately impacts hub areas. Our aim is to provide a foundation for future studies examining individual differences in network architecture and reading skill.

## Methods

### Distribution of resting-state networks across reading areas

Reading-related regions were selected on the basis of the Neurosynth meta-analytical database [22], comprising 11,406 studies as of October 31, 2017. NeuroSynth aggregates brain activation data from thousands of studies to return activation likelihood maps based on search terms. The database includes terms such as “word recognition” (74 studies), “visual word” (98 studies), and “language comprehension” (76 studies). We chose to use the term “reading” (427 studies) because it was inclusive of most of the studies returned by narrower results, and it also showed a high degree of consistency with the other terms. NeuroSynth provides two meta-analytic activation maps, one using forward-inference and the other using reverse-inference. The forward-inference map creates a map of brain areas associated with reading-related papers; the reverse-inference map returns the set of brain areas most likely to be active only in reading-related papers. The reverse-inference map thus de-weights domain-general functional areas and is more representative of reading-specific areas than the forward-inference map. Because domain-general processes are fundamental to skilled reading (especially comprehension), our primary interest was in the forward-inference map, but both maps were examined.

The 7-network cortical parcellation from Yeo and colleagues (2011) was used to represent canonical RSNs [23]. On the basis of resting-state fMRI data from 1000 subjects, this atlas identifies the following RSNs: visual, somatomotor/auditory, limbic, ventral attention, dorsal attention, default mode, and fronto-parietal. The “Liberally Masked” volumetric data was downloaded in MNI152 space and co-registered to the NeuroSynth data. For each inference map, the percentage of activation falling into each RSN (cm^3^) was calculated to provide a distribution across each RSN.

### Relationship between brain modularity and reading skill

Original data for the modularity analyses were drawn from the third and fourth waves of a larger longitudinal study (NICHD R01 HD067254, 140 children at first wave). Participants completed out-of-scanner cognitive tests and an in-scanner language task. The fMRI task also included an extensive resting-state baseline in each run, which was the primary target of analysis here. Further information about the aims of this grant can also be found elsewhere [24, 25].

**Participants** Participants were scanned in the summer and fall following completion of third or fourth grade (ages 8–11). All participants met the following criteria: native English speakers; normal hearing; normal or corrected-to-normal vision; no history of major psychiatric illness or traumatic brain injury/epilepsy; and no contraindication to MRI. Participants and their parents gave written consent to participate at the beginning of the study, with procedures carried out in accordance with Vanderbilt University’s Institutional Review Board (IRB).

Participants completed cognitive tests, including the Wechsler Abbreviated Scale of Intelligence (WASI) [26] and the Test of Word Reading Efficiency (TOWRE) [27]. Demographics and test data are summarized in Table 1.

**Table 1:** Demographics for study participants

	Grade 3	Grade 4
Participants	50	45 (15 new)
Scan runs	152	162
Gender	24 F, 26 M	23 F, 22 M
Age at scan (SD)	9.45 (0.3)	10.5 (0.3)
WASI Full-Scale IQ (SD)	113.0 (15.5)	111.0 (15.9)
TOWRE - Total Word Efficiency (SD)	109.9 (14.8)	104.6 (17.4)

**Functional MRI data** In the MRI scanner, participants performed up to four runs of a language comprehension task, which was crossed on two conditions: the modality of presentation (listening or reading) and the passage genre (expository or narrative). Each fMRI run had two baseline conditions: a modality-specific baseline task and a resting-state block with a fixation cross. The order and duration for each block varied slightly across runs but was approximately: paragraph 1 (70 s), baseline 1 (70 s), paragraph 2 (70 s), baseline 2 (70 s), and resting-state (270 s). Total scan time was 550 s for all runs, and the average amount of resting-state baseline was 272 s (4 m, 32 s) per run.

A scan run was included in the analysis only if a participant had both listening and reading scans in the same genre (e.g., auditory-expository and reading-expository). Therefore, for each year, a participant had data from either 2 or 4 scan runs (about 9 or 18 min of resting-state scan time, respectively). A scan session was excluded based on the following parameters: high-motion volumes exceeding 20%; poor task performance; and absence of a paired modality scan. In total, resting-state data from 50 children in the third wave (152 scans) and 45 children in the fourth wave (162 scans) met inclusion criteria.

**Imaging acquisition and preprocessing** All fMRI scans were acquired at Vanderbilt University Institute of Imaging Sciences on one of two Philips Achieva 3T MR scanners with a 32-channel head coil. Functional images were acquired using a gradient echo planar imaging sequence with 40 slices acquired parallel to the anterior-posterior commissure plane. Additional imaging parameters for functional images were 250 dynamics; TR = 2200 ms; TE = 30 ms; FOV = 240×240×120 mm; flip angle = 75^∘^ ; voxel size = 3×3×3.2 mm^3^.

All scans were first preprocessed using a standard pipeline in FSL (version 5.0.9) [28], and connectivity analysis was performed in the CONN toolbox [29]. fMRI data were high-pass filtered at 0.008 Hz, motion-corrected, co-registered to a structural image, normalized to MNI space and smoothed by a 5-mm FWHM spherical kernel. Outlier volumes were identified as individual fMRI volumes in which the RMS framewise-displacement exceeded 0.7. fMRI timeseries were corrected using anatCompCorr methods, which uses signal from white matter tissue and cerebrospinal fluid areas to reduce noise not related to brain activity [30]. Other covariates of no interest included six rigid motion parameters, six derivative motion parameters, and outlier volumes. Finally, we used a weighted general linear model (GLM) to model the resting block and averaged within-subject across scan sessions to get a de-noised resting-state timeseries.

**Network definition** To build off of previous work, we created networks using 264 nodes originally published by Power and colleagues (2011) [31]. The node set covers the entire brain, including subcortical areas, and has been extensively used in graph theory analyses since its publication (e.g., [32–34]). Suggested RSN assignments for each node, totaling 13 unique networks, are also available and were used to partition the network into different RSNs. fMRI timeseries correlations were calculated between each of the the 264 nodes, resulting in a single connectivity array for each subject at each time point. Matrices were then thresholded into binary maps at *r* = 0.15. (To confirm that this particular threshold did not unduly influence results, we also tested thresholds at *r* = 0.05, 0.10 and 0.20. No significant effect on the results was found.)

**Network analysis***Global modularity* (*Q*) quantifies how well the whole-brain network segregates into component RSNs. High modularity indicates that the network has much higher connectivity within RSNs compared to between RSNs; low modularity suggests that nodes do not segregate into RSNs well. For undirected and binary networks, each connection in the array is given a positive value if it links nodes in the same RSN, and it is then weighted based on the degree of each node and the total network. (For a detailed treatment, see [35].) Connection-level values were aggregated to get node-by-node and global measures of modularity for each individual, which were then mean-centered and scaled to unit variance.

A GLM was used to determine the relationship between global modularity and individual performance on the TOWRE (total word efficiency, standard score). If subject data was available at multiple timepoints (*n* = 30), it was averaged together to produce a single value. Multiple supplementary analyses were also completed to ensure that effects were not driven by cohort or motion confounds: models were also analyzed for grades 3 and 4 separately, and also when including a measure of subject motion (mean global signal change). We also examined whether there were differences in the modularity relationship between TOWRE subtests (sight word efficiency (SWE) and phonemic decoding efficiency (PDE)).

To test whether there was an RSN-level trend in the modularity-to-reading relationships, node modularity values were also investigated. For the set of nodes comprising each RSN, a one-sample *t* test was performed to see whether the RSN average was significantly greater than the global node average.

### Mapping dyslexia abnormalities onto hub areas

Two decades of neuroimaging research have allowed a relative consensus to form as to which brain regions are commonly dysfunctional in dyslexia. To determine whether there was any pattern related to network architecture in these areas, we gathered all activations from three meta-analyses comparing fMRI responses for individuals with dyslexia to typical readers [7–9]. The meta-analyses encompassed a total of 68 studies comparing dyslexic and typically developing individuals. Sample populations included children and adults under different experimental conditions. All brain areas that showed atypical activation in dyslexia (either greater or less activity) were included. When an activation spanned a large area, all reported local maxima were included.

To get measures of hubness across the brain, we used data from a connectomics study by Power and colleagues [36]. That study reports the *participation coefficient* for each of the 264 nodes previously described. The participation coefficient reflects the diversity of a node’s connectivity to different RSNs, where a higher value indicates that the node is correlated with many different RSNs. Activations from the dyslexia meta-analyses were then mapped to the geometrically closest node from this dataset, resulting in a small set of dyslexia-related nodes and a larger, unaffected set. The nodes and descriptions, along with their suggested system and atlas label, are available as an Additional file 1.

The distribution of participation coefficients across the 264 nodes was non-normal, with a large group of areas having low participation coefficients (i.e., affiliated with few RSNs) and a smaller hub-like group. Therefore, a Wilcoxon rank-sum test was performed on the participation coefficients for the two groups, which tests for the equivalence of two distributions in a non-parametric fashion.

## Results

### Distribution of resting-state networks across reading areas

**Fig. 1:**
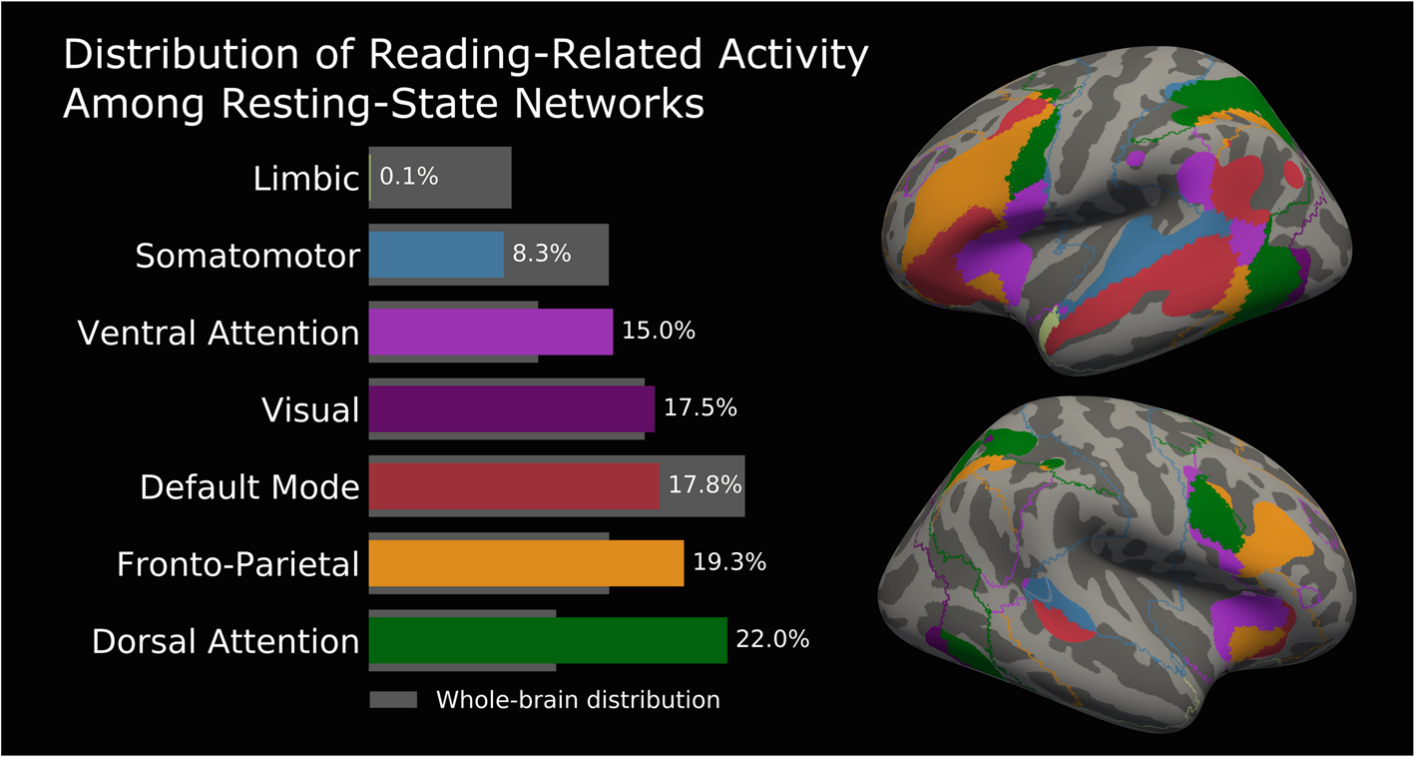
Reading areas are distributed across many resting-state networks. On the left is the volumetric breakdown of the “reading” network, pulled from a NeuroSynth automated meta-analysis (forward-inference: *p*<0.01, FDR-corrected) [22], according to the 7-network cortical parcellation from Yeo and colleagues [23]. On the right is a surface plot of the same data. Reading areas are well-distributed across different networks and load highly onto attention and executive networks. Several important reading areas, including the inferior frontal gyrus and temporo-parietal junction, sit at points where multiple networks converge, i.e., likely hub areas

A comparison of the NeuroSynth “reading” activations to the 7-network parcellation from Yeo and colleagues shows that reading is widely distributed across resting-state networks (Fig. 1). In the forward-inference map, the visual and somatomotor-auditory RSNs consituted about one quarter of the NeuroSynth activations (17.5 and 8.2%, respectively), while attention networks combined to make up 37%. The fronto-parietal (19.3%) and default mode (17.8%) networks were also highly represented. The limbic network was the only RSN which did not meaningfully overlap with the reading network. Compared to the baseline distribution of the Yeo parcellation, the visual, dorsal attention, ventral attention, and fronto-parietal networks consituted a larger portion of the activation; the limbic, somatomotor and default mode had smaller shares (Table 2).

**Table 2:** Distribution of resting-state networks across NeuroSynth maps

	Distribution across volumes (%)		
	Forward-inference	Reverse-inference	Whole-brain
Default mode	17.8	29.7	23.0
Dorsal attention	22.0	18.5	12.5
Fronto-parietal	19.3	13.3	14.7
Limbic	0.1	1.2	8.7
Somatomotor-auditory	8.3	7.6	14.7
Ventral attention	15.0	8.3	10.4
Visual	17.5	21.4	16.9
Total volume	149.9 cm^3^	106.8 cm^3^	1067.9 cm^3^

The NeuroSynth reverse-inference map was smaller than the forward-inference map by 28.7% and was restricted to areas more specific to reading. In particular, there was a higher involvement of the visual (+3.9*%*) and default mode network (+11.8*%*) compared to the forward-inference map. On the other hand, networks supporting domain-general functions (dorsal attention, ventral attention, fronto-parietal) were relatively less present (−3.4, −6.7, and −6.0*%*, respectively). Representation of the somatomotor-auditory and limbic network involvement was mostly unchanged.

### Relationship between brain modularity and reading skill

**Fig. 2:**
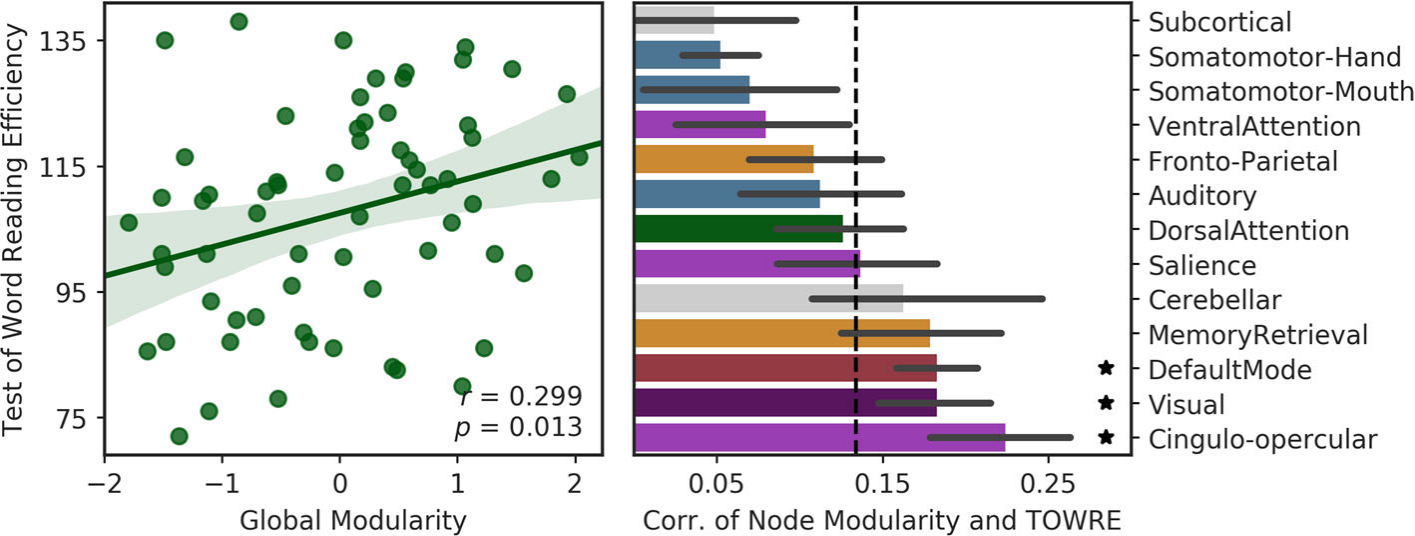
Modularity metrics at rest predict reading skill. Global modularity, the degree to which a whole-brain network separates into RSNs, was positively related to reading skill across all subjects (*N*=65). Modularity for individual nodes was also positive overall (*r*_avg_=0.134), but was significantly higher for nodes in the visual, default mode and cingulo-opercular RSNs (*p*<0.01). RSN colors correspond to the dominant Yeo RSN displayed in Fig. 1

Individual differences in global modularity were predictive of TOWRE - Total Word Efficiency standard scores (Fig. 2). Modularity had a significant positive relationship with out-of-scanner reading metrics (for all subjects: *r*=0.299, *p*=0.013). This effect was also significant when data was analyzed separately by grade (*r*_*G*3_=0.333, *p*=0.014; *r*_*G*4_=0.359, *p*=0.012). The two subtests that constitute the TOWRE both showed a positive relationship with global modularity, as well (*r*_PDE_=0.314, *p*=0.009; *r*_SWE_=0.251, *p*=0.039).

When examined at the individual node level, the average correlation between modularity and TOWRE scores was 0.134. Three RSNs had average node correlations that were significantly higher than this global network mean: the default mode (*r*=0.183, *p* <0.001), visual (*r*=0.183, *p*=0.004) and cingulo-opercular networks (*r*=0.224, *p* <0.001) (Fig. 2).

### Mapping dyslexia abnormalities onto hub areas

**Fig. 3:**
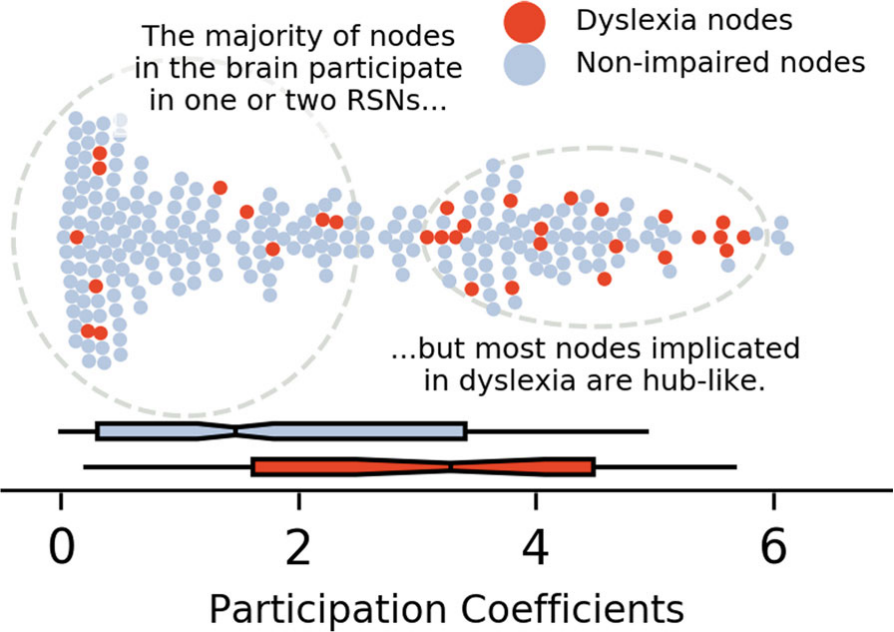
Dyslexia disproportionately impacts hub areas. Among the brain areas examined in Power and colleagues [36], nodes implicated in dyslexia have higher participation coefficients (32 nodes) compared to the rest of the brain (232 nodes)

Across the three meta-analyses, 32 of the 264 nodes showed abnormal functioning in dyslexia. Figure 3 shows the node-by-node distribution of participation coefficients for the entire set. The median participation coefficient for unaffected nodes was 1.47; for dyslexia-related nodes it was 3.28. A Wilcoxon rank-sum test between the dyslexia and unaffected nodes showed that dyslexia affects brain areas with higher participation coefficients than would otherwise be expected (*U*=4946.0, *p* <0.001).

Table 3 presents data for nodes that were identified in at least two of the three meta-analyses, along with nearby Talairach atlas labels. These six areas are distributed across the brain and are located near major regions of previous investigation. Each of these nodes is in the top half of the distribution of all participation coefficients, with the occipito-temporal node (near the visual word form area) having the lowest participation coefficient rank (53rd percentile) and the node near the pars orbitalis of the inferior frontal gyrus having the highest (97th percentile).

**Table 3:** Participation coefficients (PC) for nodes identified in multiple dyslexia meta-analyses

Atlas label	MNI coordinates			Hubness		Suggested network	
	X	Y	Z	PC	%	Yeo 2011	Power 2011
L BA 47, L insula	− 35	20	0	5.46	97.7	Fronto-parietal	Salience
L mid. occ. gyrus	− 42	− 60	− 9	5.35	97.0	Dorsal attn.	Dorsal attn.
L putamen	− 15	4	8	3.20	67.4	–	Subcortical
L inf. par. lobule	− 53	− 49	43	3.07	65.5	Default mode	Fronto-parietal
L sup. temp. gyrus	− 55	− 40	14	2.94	64.0	Ventral attn.	Ventral attn.
L fusiform gyrus	− 47	− 51	− 21	1.66	50.8	Dorsal attn.	Uncertain

## Discussion

The current study sought to determine whether aspects of the brain’s network architecture are related to reading. The results suggest that an efficient network organization, i.e., one in which brain areas form RSNs, is important for skilled reading, and that dyslexia can be characterized by abnormal functioning of hub regions that map information between multiple systems. To our knowledge, this is the first time the relationship between modularity and hubness to reading skill has been described, adding to a foundation of work built on other connectivity methods.

A connectomics approach to reading illuminates—not displaces—previous neuroimaging research, much of which focused on localizing specific cognitive processes. One insight is that much of the “reading network” falls in domain-general RSNs such as the attention and executive networks (see Fig. 1). While these areas perform a specific function in reading, they are also often involved in other processes. For example, the dorsal attention network (DAN) encompasses the visual word form area, an area that has been the subject of much interest and debate in reading and dyslexia research [37]. It is probable that this area is so important in reading not only because it is connected to language areas [38], but also because it is tightly tied to other areas that control goal-directed attention [39]. Koyama et al. found that children with a historical diagnosis of dyslexia had persistent de-coupling of the DAN compared to typical readers regardless of remediation status [40]. Vogel et al. found that reading ability in typical children and adults (including decoding and passage comprehension ability) predicted increased correlations between the visual word form area and the DAN [41]. The nesting of this orthographic-processing area within the DAN is thus important to its role in reading.

Our data support a hypothesis that high “intrinsic” efficiency within RSNs is important even for relatively specific tasks such as word recognition. This hypothesis is broadly consistent with frameworks such as “interactive specialization,” in which a more efficient and specialized visual recognition system would have higher connectivity between visual areas, in turn leading to higher modularity in the RSN [42]. The relationship between reading and modularity was particularly high in the visual, default mode, cingulo-opercular networks. It is not yet possible to say whether modularity within these specific RSNs correlates most highly with reading because of their functional roles in reading processes or whether they simply capture global trends better than other networks. There is some reason to suspect specificity, however. In studies of remediation-induced changes to connectivity, increased connectivity within the visual network [40] and cingulo-opercular network [43] have predicted reading improvement in dyslexic children. Future work will need to examine not just the intrinsic connectivity, but the relationships between these networks during reading tasks. The default mode network, for example, is typically anti-correlated with “task-positive” networks such as the fronto-parietal network. A high degree of anti-correlation has been reported to be important for performance on a variety of cognitive processes [44, 45], but recent work suggests that high modularity and connectivity of the default mode during higher-level cognition is fundamental to processes relying on self-referential and memory retrieval processes, such as those found in language [46]. The dynamics behind these interactions will be important for further establishing a framework for investigating the roles of specific networks during reading.

The additional findings that hubs areas are key in dyslexia are not surprising: dyslexia has often been thought to be a disorder related to combining information across different functional systems, and in the context of connectomics, hub areas play this privileged role. For example, the posterior temporal sulcus connects visual and auditory networks by binding letters to sounds [47, 48] and the inferior frontal gyrus has many different subdivisions supporting language parsing and manipulation [49]. However, casting dyslexia dysfunction into a connectomics perspective opens up new hypotheses and research avenues. For example, the brain areas of interest and neuroimaging metrics can be unified across other developmental disorders, including ADHD, specific language impairment, and autism [18]. Another benefit is that it opens up many more avenues for investigating dyslexia using functional and diffusion MRI, which can be performed in younger children and without administering a cognitive task.

A caveat with connectomics analyses, including those presented here, are that results for the modularity analyses are often based on RSN parcellations from previous literature [31]. However, brain organization changes throughout development, with several studies suggesting that at least some RSNs become less modular with age [11]. Therefore, it is an open question as to how network architecture develops over time, and how best to measure it is under active investigation. Disentangling this complex interchange between development, experience and network architecture will be an important future goal.

### Conclusions

Fluent readers must be masters of many cognitive processes and efficiently pass information between the brain areas that subserve them. Since these areas are distributed throughout the brain and across functional subdivisions, having an efficient network architecture is likely to make the reading processes more rapid and precise. The results presented here support this hypothesis by showing that intra-connectivity within RSNs is correlated with reading skill, and that abnormalities in dyslexia localize onto the hub areas that connect RSNs. Overall, use of a connectomics framework opens up new possibilities for investigating reading difficulty, especially its commonalities across other neurodevelopmental disorders.

## Additional file

Additional file 1 Details for 264 nodes used in graph theory analyses. Table of node coordinates, community assignments, network measures and implication in the three dyslexia meta-analyses. Atlas labels were assigned using the Talaraich Daemon atlas. (CSV 20 kb)
